# A Novel CIP2A and BCL-XL Clinical Diagnostic Toolkit to Predict Disease Progression and Treatment-Free Remission in Chronic Myeloid Leukaemia

**DOI:** 10.3390/ijms27072991

**Published:** 2026-03-25

**Authors:** Ammar A. Basabrain, Gemma M. Austin, Alison K. Holcroft, Jane F. Apperley, Richard E. Clark, Shankar Varadarajan, Claire M. Lucas

**Affiliations:** 1 Department of Medical Laboratory Sciences, Faculty of Applied Medical Sciences, King Abdulaziz University, Jeddah 21589, Saudi Arabia; 2 Hematology Research Unit, King Fahd Medical Research Centre, King Abdulaziz University, Jeddah 21589, Saudi Arabia; 3 Department of Molecular and Clinical Cancer Medicine, University of Liverpool, Liverpool L69 3GA, UK; 4 Centre for Haematology, Imperial College London at Hammersmith Hospital, London W12 0HS, UK; 5 Chester Centre for Leukaemia Research, Chester Medical School, University of Chester, Bache Hall, Chester CH2 1BR, UK

**Keywords:** BCL-XL, CIP2A, CML, imatinib, dasatinib, SPIRIT2, DESTINY, clinical trial, BCR::ABL1, treatment failure, molecular responses (EMR, MR2 and MR3), treatment-free remission (TFR), biomarker

## Abstract

Biomarkers that predict disease progression and treatment-free remission (TFR) would be of significant clinical value in chronic myeloid leukaemia (CML). We have previously shown that CIP2A levels at diagnosis can identify patients at increased risk of progression. One mechanism by which CIP2A acts is through upregulation of the anti-apoptotic gene BCL-XL. In this study, we evaluated BCL-XL mRNA expression as a diagnostic biomarker using samples from the SPIRIT2 and DESTINY clinical trials. In SPIRIT2, which compared imatinib and dasatinib as first-line therapies, high BCL-XL expression was associated with treatment failure, poor early molecular response, and lower rates of MR2 and MR3 achievement in patients treated with imatinib. In the DESTINY trial, which assessed treatment de-escalation and discontinuation, BCL-XL expression was significantly higher in patients who experienced molecular relapse compared to those achieving sustained TFR. Notably, increases in BCL-XL were detectable 6 to 8 months prior to molecular relapse, suggesting it may serve as an early biomarker of unsuccessful TFR. We now propose a clinical diagnostic toolkit combining CIP2A and BCL-XL biomarkers to stratify CML patients by the risk of disease progression and likelihood of achieving successful TFR.

## 1. Introduction

Tyrosine kinase inhibitors (TKIs) have substantially increased the survival rate of patients with chronic-phase chronic myeloid leukaemia (CML) to nearly 90%, leading to a near-normal life expectancy [1]. However, two distinct clinical challenges remain. Firstly, a number of patients will encounter treatment failure, and some will progress to blast crisis, with a 10% survival rate [2,3,4]. Patients who have treatment failure will require a change in therapy in order to prevent disease progression [5,6,7]. Secondly, although TKIs are well-tolerated, 30% of patients have side effects that interfere with their quality of life [8,9,10]. Treatment-free remission (TFR) [11,12,13] is an option for those patients who achieve an optimal response, especially as successful achievement of sustained TFR may enhance their quality of life [6,14]. Although the depth and duration of molecular response are associated with the subsequent success of a TFR attempt, these correlations are not reliable enough to prospectively identify patients who can successfully stop treatment. Currently, only serial molecular monitoring during initial dose reduction reliably predicts recurrence after TKI cessation [15].

A number of scoring systems have been established to identify groups of patients at high risk of disease progression [16,17,18]. However, long-term imatinib treatment data from Italy suggest that over 70% of patients classified as high risk by Sokal or other scoring approaches continue to have optimal outcomes at 7 years [19]. The EUTOS long-term survival (ELTS) score was recently introduced to evaluate the risk of disease progression. In the high-risk ELTS category, only 7% of CML patients died from their disease over an eight-year period [20]. These observations restrict the application of these scoring systems in shaping treatment choices for individual patients in the clinic.

Recently, we have demonstrated that diagnostic CIP2A protein levels are a biomarker for predicting treatment failure and disease progression in the UK SPIRIT2 trial [21]. Our team has previously established a connection between CIP2A and BCL-XL, showing that CIP2A shifts the apoptotic balance toward an anti-apoptotic phenotype, leading to the upregulation of the anti-apoptotic protein BCL-XL [22]. Mechanistically, CIP2A is an endogenous inhibitor of PP2A, and PP2A functions as a key negative regulator of oncogenic signalling [23]. Therefore, CIP2A-mediated PP2A inhibition provides a biologically plausible route to sustained survival signalling, including STAT5-dependent transcriptional upregulation of BCL-XL in BCR::ABL1-positive cells [22,24,25]. BCL-XL plays a significant role in the progression of CML [26]. In both CML blast crisis cells (K562) and AML cells carrying *BCR::ABL1* genes (HL-60/*BCR::ABL1*), changes in the expression levels of BCL-2 and BCL-XL occur [27]. This elevation in BCL-XL expression hinders the release of cytochrome c from mitochondria. BCR::ABL1 stimulation through STAT5 triggers the heightened expression of BCL-XL in these cells [24,28,29]. BCL-XL is essential for the survival of progenitor cells in CML blast crisis [30]. In a study involving a BCL-XL-deficient mouse model, disease progression was significantly impeded, indicating that BCL-XL is necessary for both the progression of CML and the survival of leukemic progenitor cells. Additionally, BCL-XL can obstruct pro-apoptotic stimulation in a process that may be dependent on or be independent of BCR::ABL1 kinase activity [31,32,33]. In primary CD34+ stem cells obtained from patients with high CIP2A levels, TKIs did not induce significant apoptosis. However, A-1331852, a selective BCL-XL inhibitor [34], triggered apoptosis at low nM concentrations with short-term exposure [22]. In contrast, A-1331852 had no impact on the survival of mononuclear cells extracted from healthy volunteers, suggesting that targeting BCL-XL could be a novel therapeutic strategy in CML [22]. Based on prior functional evidence supporting CIP2A–PP2A–STAT5–BCL-XL regulation, we hypothesised that diagnostic BCL-XL expression would associate with clinically relevant outcomes. Building on the strong association between CIP2A and BCL-XL that we previously reported [22], we investigated the prospective prognostic value of BCL-XL mRNA in newly diagnosed CML patients enrolled in the SPIRIT2 clinical trial, and a subset of patients enrolled in the DESTINY trial attempting TFR.

## 2. Results

All 159 SPIRIT2 samples were deemed suitable for BCL-XL expression assessment. Their patient characteristics are detailed in Table 1A. In summary, 81 patients were treated with imatinib, and 78 received dasatinib. A total of 18 patients progressed to blast crisis, with 9 from the imatinib group and 9 from the dasatinib group. The median age in both treatment arms was 53 years. The sample subset from TFR patients comprised 24 local samples from DESTINY entrants along with two additional patients who discontinued therapy outside the trial (Table 1B).

### 2.1. SPIRIT2 Trial

#### 2.1.1. BCL-XL Expression and Established Scoring Systems

**Figure 1:**
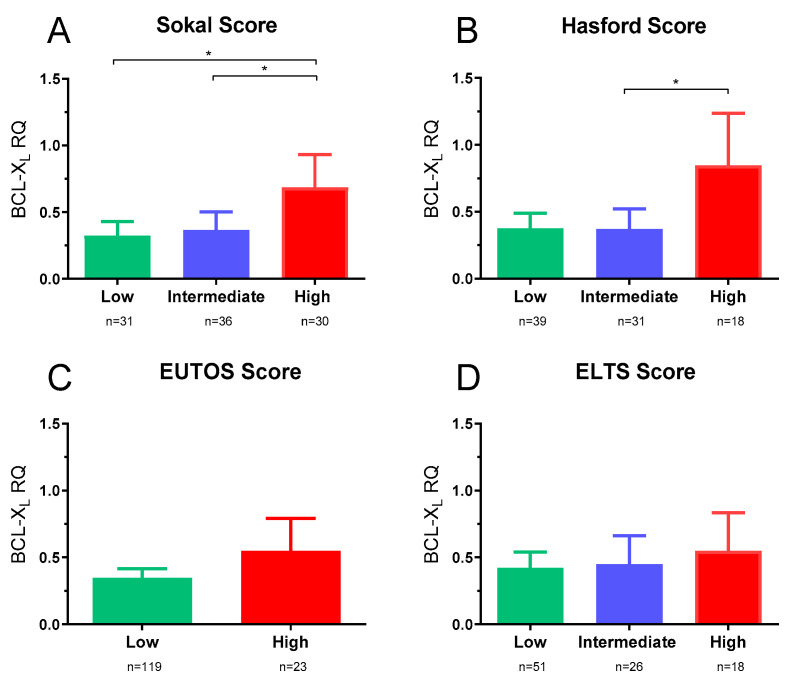
BCL-XL mRNA expression levels stratified by various CML scoring systems for SPIRIT2 CML patients. BCL-XL mRNA expression SPIRIT2 CML stratified by categories of (**A**) the Sokal score (low vs. high, *p* = 0.022, and intermediate vs. high, *p* = 0.018), (**B**) the Hasford risk score (intermediate vs. high, *p* = 0.04), (**C**) the EUTOS score, and (**D**) the ELTS score. Statistical analysis performed by GraphPad Prism Version 9 using Kruskal–Wallis test, and Mann–Whitney U test. (Error bars represent standard error of the mean (S.E.M). *p*-values specified where significant, * = *p* ≤ 0.05).

BCL-XL mRNA expression was stratified using the four various CML scoring systems’ categorical-risk categories. The components of the various scoring systems were not required to be recorded at trial entry; therefore, 39% (Sokal), 44% (Hasford), 10% (EUTOS), and 39% (ELTS) could not be assigned a score. The results revealed significant differences in BCL-XL mRNA expression between the categorisation groups for both the Sokal and Hasford scores. BCL-XL expression was considerably greater in the high-risk Sokal group than in the intermediate-risk group (Figure 1A; *p* = 0.018) and the low-risk group (*p* = 0.022). No significant variations in BCL-XL expression were seen between the low- and intermediate-risk groups. Using the Hasford score, the high-risk group had a greater BCL-XL expression than the intermediate-risk group (Figure 1B; *p* = 0.04). No significant differences in BCL-XL expression were seen between the low-risk and intermediate-risk groups, nor between the low-risk and high-risk groups (Figure 1B). For the EUTOS and ELTS scores, there were no significant differences in BCL-XL expression between the various categories for either score, as shown in Figure 1C,D.

#### 2.1.2. High BCL-XL Expression Levels Are Associated with Treatment Failure in SPIRIT2 Samples

The relationship between BCL-XL expression and OS, PFS and FFP was examined by assigning the lowest and highest quartiles of mRNA expression to categorise patients into high and low BCL-XL groups (Supplementary Figure S1). This was carried out separately for patients receiving imatinib, those receiving dasatinib and for both groups combined. In all of these groups, BCL-XL expression was not associated with OS, PFS or FFP (Supplementary Figure S2).

**Figure 2:**
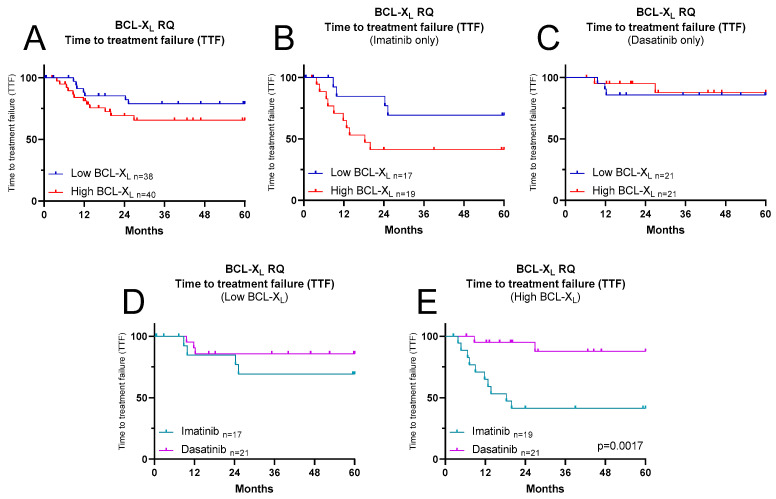
Kaplan–Meier curves illustrating the time to treatment failure (TTF) for SPIRIT2 patients, stratified by diagnostic BCL-XL mRNA expression levels. (**A**) Comparison of high and low BCL-XL mRNA expression among all SPIRIT2 samples studied. (**B**) Comparison of high and low BCL-XL mRNA expression levels in SPIRIT2 imatinib recipients, and (**C**) in dasatinib recipients. (**D**) Comparison of patients with low BCL-XL mRNA expression according to drug received, and (**E**) similarly for those with high BCL-XL mRNA expression. Statistical analysis was performed by GraphPad Prism Version 9 using the Log-rank (Mantel–Cox) test; *p*-values are shown where significant.

An analysis of BCL-XL mRNA expression in relation to time to treatment failure (TTF) revealed no significant differences. However, there was a trend for a higher rate of treatment failure in patients with high BCL-XL expression compared to those with low expression (Figure 2A). When stratified by TKI treatment, this difference was primarily observed among imatinib recipients (Figure 2B,C). TTF for imatinib and dasatinib was analysed separately for patients with low and high BCL-XL expression. For patients with low BCL-XL expression, there was a non-significant trend for imatinib recipients to have a higher rate of treatment failure (Figure 2D), and a similar finding was seen in patients with high BCL-XL expression, this time achieving statistical significance (Figure 2E; *p* = 0.0017). These findings suggest that treatment failure events were primarily driven by imatinib recipients and predominantly occurred in patients with high BCL-XL expression.

#### 2.1.3. High BCL-XL Expression Levels Are Associated with Delay in Time to Molecular Response

**Figure 3:**
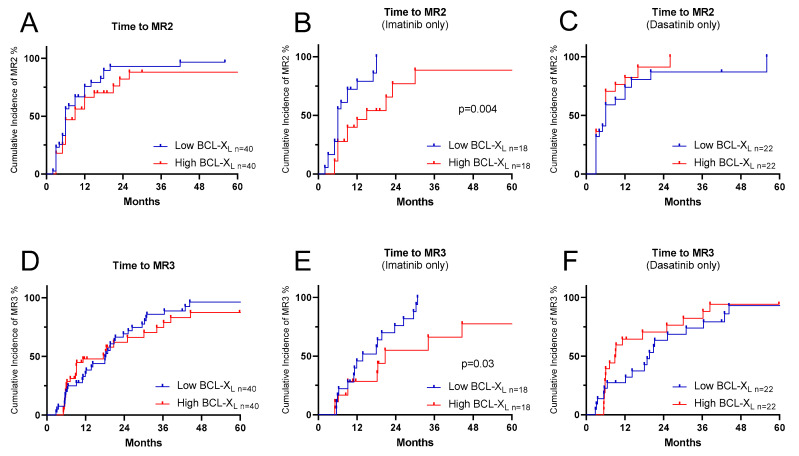
Cumulative incidence of time to molecular responses (MR2 and MR3) for SPIRIT2 CML patients, stratified by their diagnostic BCL-XL mRNA expression levels. Panels (**A**–**C**) show Kaplan–Meier curves for time to MR2, while panels (**D**–**F**) show Kaplan–Meier curves for time to MR3.

In the SPIRIT2 samples, we examined the association between BCL-XL expression levels and time to various molecular response levels. Overall, no association was observed between low and high BCL-XL expression and early molecular response (EMR) rates, though for those patients who received imatinib, high BCL-XL expression conferred a lower EMR rate (21%) than in those with low BCL-XL expression (79%; Supplementary Table S1; *p* = 0.03). Similarly, in imatinib- but not dasatinib-treated patients, high BCL-XL expression was correlated with a lower probability of attaining MR2 (*p* = 0.004, Figure 3B) and MR3 (*p* = 0.03, Figure 3E). The median time for achieving MR2 was 6 months for patients with low BCL-XL expression and 15 months for those with high BCL-XL expression. Similarly, the median time to achieve MR3 was 14 months for patients exhibiting low BCL-XL expression and 21 months for those with high BCL-XL expression. To include patients outside the pre-specified extreme-quartile comparison (Q1 vs. Q4), we performed analysis of the intermediate group (Q2–Q3) for time to MR2 and MR3 (Supplementary Figure S3). In MR4 and MR4.5, the trend observed in imatinib-treated patients persisted. However, no significant association was found between low and high BCL-XL expression levels in MR4 and MR4.5. Notably, patients with low BCL-XL expression showed a higher probability of achieving MR4 and MR4.5 compared to those with high BCL-XL expression (Supplementary Figure S4). No correlation between BCL-XL and time to EMR, MR2 or MR3 was seen for dasatinib recipients.

To assess whether BCL-XL expression independently predicted the clinical outcome after adjustment for relevant covariates, multivariable Cox regression analysis was performed. High BCL-XL expression remained independently associated with a shorter time to treatment failure after adjustment for treatment, age, and gender (HR = 2.51, 95% CI 1.34–4.70, *p* = 0.004), and was also significantly associated with the time to MR2 (HR = 1.92, 95% CI 1.15–3.21, *p* = 0.013) and time to MR3 (HR = 1.73, 95% CI 1.05–2.84, *p* = 0.030) (Supplementary Table S2). In stratified analyses, these associations were most pronounced in imatinib-treated patients, in whom high BCL-XL expression was associated with an increased risk of treatment failure (HR = 4.03, 95% CI 1.67–9.73, *p* = 0.002) and remained significantly associated with the time to MR2 (HR = 2.78, 95% CI 1.41–5.48, *p* = 0.003) and time to MR3 (HR = 2.26, 95% CI 1.16–4.41, *p* = 0.017). By contrast, no significant associations were observed in dasatinib-treated patients for TTF, MR2, or MR3 (Supplementary Table S2). These data support our observation that BCL-XL is an independent prognostic factor. In matched samples, CIP2A RQ and BCL-XL RQ were not significantly correlated (Supplementary Figure S5).

The findings from the BCL-XL data in the SPIRIT2 trial suggest that high BCL-XL expression correlates with poorer patient outcomes, particularly in terms of treatment failure and the time required to achieve molecular responses in imatinib-treated patients. As a result, BCL-XL expression could potentially serve as a biomarker for predicting treatment failure and molecular response outcomes, including EMR, MR2, and MR3, in patients treated with imatinib and motivate replication in additional datasets. However, this trend was not evident in dasatinib-treated patients, possibly because dasatinib is more effective in suppressing BCL-XL than imatinib [35,36]. Consequently, CIP2A may be a more suitable biomarker for treatment failure [21].

### 2.2. DESTINY Trial

#### 2.2.1. BCL-XL mRNA Expression in Patients Attempting TFR

**Figure 4:**
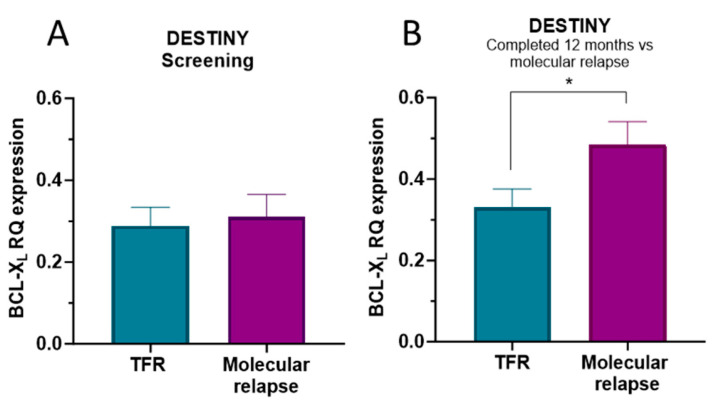
BCL-XL mRNA expression in the DESTINY trial, comparing patients who achieved TFR and those who experienced a molecular relapse. (**A**) At the screening stage for trial entry (beginning of the de-escalation phase), and (**B**) at the completion of 12 months or at the time of relapse within the 12-month period (12 months vs. relapse; *p* = 0.02). Statistical analyses were conducted with GraphPad Prism Version 9.5, using the Mann–Whitney U test. *p*-values represented with asterisks where significant; * = *p* ≤ 0.05.

At DESTINY trial entry, there was no significant difference in BCL-XL mRNA expression between patients who went on to achieve TFR and completed the study and those who experienced molecular relapse (Figure 4A,B). Interestingly, when we looked at samples taken 12 months into the trial for patients who subsequently achieved TFR and at molecular relapse, this analysis revealed significant differences in BCL-XL mRNA expression between these groups of patients. Specifically, BCL-XL mRNA expression was higher in patients experiencing molecular relapse compared to those who did not relapse and achieved TFR (Figure 4B; *p* = 0.02). However, this observation was not clinically useful, as we were already aware of the molecular relapse status of these patients at the time of analysis.

**Figure 5:**
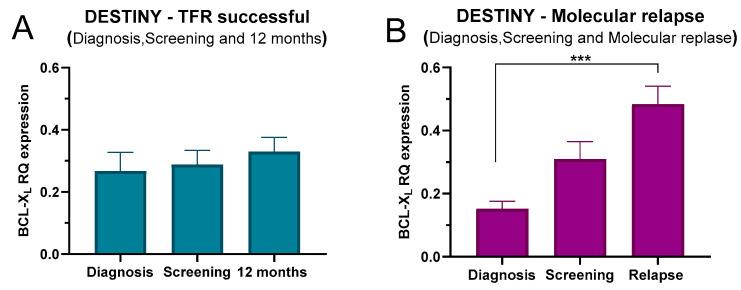
BCL-XL mRNA expression in patients attempting TFR. (**A**) BCL-XL mRNA expression levels in patients who successfully achieved TFR N = 12. (**B**) BCL-XL mRNA expression levels in patients who experienced a molecular relapse (*p* = 0.0014), with a specific emphasis on the diagnosis and relapse stages (diagnosis vs. relapse; *p* = 0.0009) N = 8. Statistical analyses were conducted with GraphPad Prism Version 9.5, using Kruskal–Wallis test, and Dunn’s multiple comparison test, *p*-values represented with asterisks where significant; *** = *p* ≤ 0.001.

Subsequently, BCL-XL mRNA expression was analysed across three distinct stages within each DESTINY outcome group, diagnosis, trial entry and 12 months (for patients who achieved TFR) or at molecular relapse. In patients who achieved TFR, no differences were detected in BCL-XL mRNA expression between the diagnosis, trial entry, and 12-month stages (Figure 5A). In contrast, a significant increase in BCL-XL mRNA expression was observed across the three stages in patients who experienced a molecular relapse (Figure 5B; *p* = 0.0014), with multiple comparison tests revealing that this significance was notably observed between the diagnosis and relapse stages (Figure 5B) (diagnosis vs. relapse stages; *p* = 0.0009).

#### 2.2.2. BCL-XL Expression Is Elevated During the De-Escalation Phase for Molecular Relapse Patients

**Figure 6:**
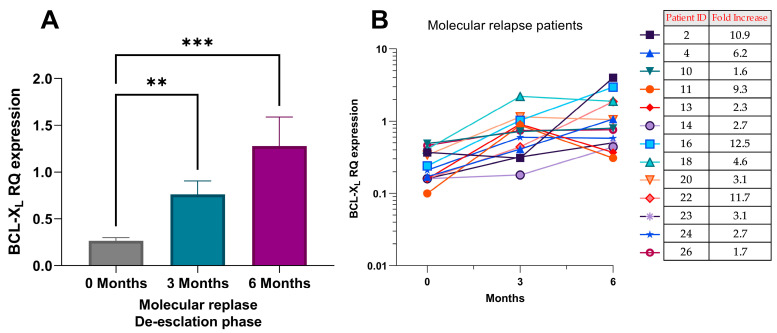
BCL-XL mRNA expression in patients experiencing molecular relapse in the DESTINY trial. (**A**) Expression at 0, 3, and 6 months after entering the de-escalation phase. The analysed time points are 0 (trial entry), 3, and 6 months following the beginning of the de-escalation phase (0 vs. 3 months; *p* = 0.003, and 0 vs. 6 months; *p* = 0.0002). Statistical analysis was conducted using GraphPad Prism Version 9.5, employing the Friedman test and Dunn’s multiple comparison test. *p*-values are indicated where significant, ** = *p* ≤ 0.01, *** = *p* ≤ 0.001. (**B**) Individual trends in BCL-XL mRNA expression in the 13 patients who experienced a molecular relapse during the DESTINY trial. Measurement at 0, 3, and 6 months following the beginning of the de-escalation phase. The data have been presented with the respective patient numbers listed in Table 1B and their corresponding fold changes.

The finding that BCL-XL mRNA expression was significantly higher at molecular relapse had no additional clinical value as molecular relapse would already have been detected by routine molecular monitoring. We next measured BCL-XL expression during the first 6 months of the de-escalation. In total, 13 patients who relapsed during the trial were studied, with three BCL-XL expression measurements collected for each patient at different time points during the de-escalation phase. These time points included trial entry (0 months), 3 months after entry (3 months), and 6 months after entry (6 months) into the de-escalation phase. Significant differences in BCL-XL mRNA expression were observed at the three time points for patients who relapsed (Figure 6A; *p* = 0.0002). Multiple comparison tests revealed a significant, gradual increase in BCL-XL mRNA expression in patients who relapsed, between 3 and 6 months into the de-escalation phase, as illustrated in Figure 6A (0 months vs. 3 months, *p* = 0.003; 0 months vs. 6 months, *p* = 0.0002). The change between 3 and 6 months was not statistically significant. This finding is particularly important as the increase in BCL-XL expression occurred at least 6–8 months before the actual molecular relapse was detected in the laboratory (before BCR::ABL1 recurrence or loss of MMR (BCR::ABL1 ratio ≥ 0.1%)). These results suggest that changes in BCL-XL expression during the de-escalation treatment phase could serve as a biomarker for molecular relapse.

We next examined the BCL-XL mRNA expression fold change in 13 patients who experienced a molecular relapse (Figure 6B and Supplementary Table S3). Fold changes observed increased within the first 6 months following trial entry. Most molecular relapse patients exhibited a rise in BCL-XL mRNA expression between 0 and 3 months, with one exception: a patient who demonstrated an increase between 3 and 6 months. The average fold increase was observed to be 5.6-fold. Fold increases ranged from a minimum of 1.6-fold to a maximum of 12.5-fold, as illustrated in Figure 6B. These calculations were based on the lowest and highest BCL-XL mRNA expression levels recorded at the three time points. These findings suggest that increases in BCL-XL expression could potentially serve as a biomarker for patients experiencing molecular relapse during TFR.

### 2.3. Applying CIP2A and BCL-XL Biomarkers to Clinical Practice to Prevent Disease Progression and Achieve TFR

Three important clinical challenges remain for the treatment and management of CML patients. Firstly, can we reliably predict patients at diagnosis who will progress into blast crisis? At the opposite end of the clinical scale are a group of patients who respond well to TKIs and achieve a deep molecular response but will remain on treatment indefinitely. Secondly, can we predict those patients for whom it is safe to stop their treatment? Thirdly, can we identify novel therapeutic targets to eliminate the residual LSC?

**Figure 7:**
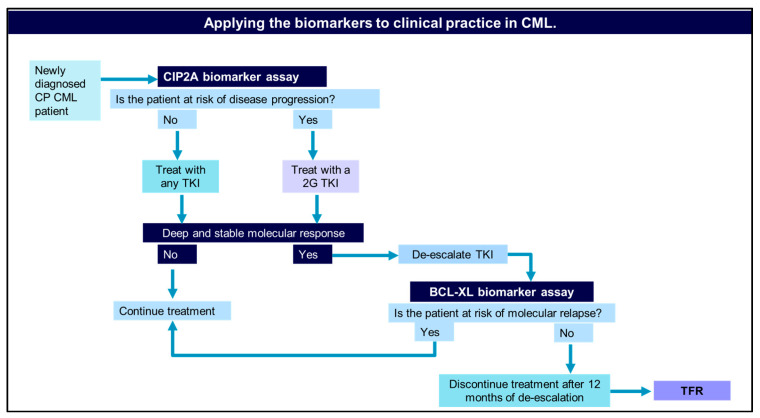
Applying CIP2A and BCL-XL biomarkers to clinical practice in CML. A biomarker-driven approach to the treatment and management of chronic-phase (CP) chronic myeloid leukaemia (CML) patients. The decision-making process begins with a CIP2A biomarker assay, which stratifies patients based on their risk of disease progression. Low-risk patients (low CIP2A) can be treated with any tyrosine kinase inhibitor (TKI), while high-risk patients (high CIP2A) receive a second-generation (2G) TKI. Patients who achieve a deep and stable molecular response are considered for TKI de-escalation. The BCL-XL biomarker assay is then used to assess the risk of molecular relapse following de-escalation. Patients with a high risk of relapse (high BCL-XL) continue TKI therapy, whereas those with a low risk (low BCL-XL) can discontinue treatment after 12 months of de-escalation, achieving treatment-free remission (TFR).

We have validated CIP2A as a biomarker of disease progression both in a local cohort of patients and in the SPIRIT2 clinical trial [21,37,38]. In this study, we have reported that BCL-XL is a biomarker to identify patients at risk of molecular relapse following treatment discontinuation. Now, we can bring these two biomarkers together in a clinical toolkit to prevent blast crisis and allow patients to achieve TFR. We suggest that for a newly diagnosed patient, you should perform a CIP2A biomarker assay. This will determine if a patient is at risk of disease progression. The results will inform the clinician as to what treatment to use. Following treatment, the aim is to achieve a deep and stable molecular response. Once in a deep and stable molecular response, then the patient may attempt to discontinue treatment by dose de-escalation [39]. At the start of the de-escalation phase and at 3 or 6 months during de-escalation, measuring changes in BCL-XL expression levels can determine if a patient is at risk of molecular relapse. If the patient is at risk of molecular relapse, then they should continue TKI treatment. If a patient has no risk of molecular relapse, then this patient should continue the de-escalation phase for a total time of 12 months and then stop TKI treatment, leading to a successful treatment-free remission (Figure 7). Future work will be to investigate this further using the whole DESTINY clinical trial.

## 3. Discussion

The examination of the BCL-XL mRNA expression in the SPIRIT2 study reveals that patients with high-risk Sokal and Hasford scores exhibited higher BCL-XL expression compared to those in intermediate- and low-risk categories. Furthermore, the findings suggest a connection between increased BCL-XL expression and treatment failure, as well as poorer EMR, MR2, and MR3 for patients receiving imatinib. BCL-XL expression did not show a clear predictive value for dasatinib-treated patients.

BCL-XL plays a key role in megakaryocyte differentiation, proliferation, and platelet survival, as has been extensively reviewed [40,41,42]. BCL-XL is essential for platelet survival and is known to degrade in aged platelets, rendering them susceptible to apoptosis [42]. The use of BCL-XL inhibitors, such as ABT-737, has demonstrated a dose-dependent decrease in platelet half-life and consequent thrombocytopenia, with aged platelets showing greater sensitivity to the inhibitor compared to newly synthesised platelets [43,44]. In the SPIRIT2 study, high BCL-XL expression was observed in patients categorised as high-risk according to the Sokal and Hasford scores but not the EUTOS and ELTS scores. This discrepancy could be due to differences in the scoring system equations, with Sokal and Hasford scores incorporating the platelet count as a parameter, while ELTS and EUTOS scores do not (Supplementary Table S4). Consequently, the platelet count impacts the risk score in the Sokal and Hasford systems, with a direct proportion between the platelet count and risk values. In contrast, ELTS scores exhibit an inverse relationship between the platelet count and risk values, and EUTOS does not include the platelet count in its formula.

Several studies have reported a considerable percentage of patients discontinuing imatinib treatment due to treatment failure, toxicity, and unsatisfactory responses [45,46,47]. These findings highlight the need to identify a measurable biomarker to predict the long-term effects of imatinib treatment in CML patients. Our analysis of the SPIRIT2 trial supports the potential of BCL-XL expression as such a biomarker, as it is associated with inferior outcomes in imatinib-treated patients.

High BCL-XL expression showed an association with an inferior molecular response in imatinib-treated patients. Despite extended follow-up periods, many patients do not achieve MMR, and our analysis suggests that BCL-XL expression may help predict which imatinib-treated patients are unlikely to achieve MMR. Furthermore, in vitro studies have demonstrated that imatinib-resistant CML cells exhibit high BCL-XL expression [27], and BCL-XL inhibitors selectively promote apoptosis in imatinib-resistant CML cells, blast crisis CML cells, and CD34+ progenitor cells, without affecting normal mono-nuclear cells [22,48]. The presence of minimal resistant cells or CD34+ progenitor cells resistant to imatinib could affect the time it takes to achieve a molecular response. Consequently, high BCL-XL expression may influence the molecular response in imatinib-treated patients. These findings underline the potential of BCL-XL expression as a predictor of long-term imatinib treatment failure, which could help guide clinical decision-making for optimal patient outcomes.

The collective evidence from these studies suggests that BCL-XL expression could be a valuable biomarker in predicting imatinib treatment failure and the long-term impact of imatinib on CML patients. A better understanding of BCL-XL expression patterns in CML patients could facilitate the identification of patients who may require alternative treatment strategies or closer monitoring. Furthermore, the development of targeted BCL-XL inhibitors may provide an additional therapeutic approach to overcoming resistance and improving outcomes in CML patients with high BCL-XL expression. In summary, BCL-XL expression appears to play a significant role in determining the effectiveness of imatinib treatment in CML patients. High BCL-XL expression is associated with an inferior molecular response and treatment failure. Accordingly, this highlights the need for further research to validate BCL-XL as a predictive biomarker and explore its potential in guiding personalised treatment approaches. By leveraging this knowledge, clinicians could optimise treatment strategies for CML patients, ensuring that those with high BCL-XL expression receive appropriate interventions to improve their treatment plan. While BCL-XL may offer some additional clinical information, it does not predict disease progression, and thus CIP2A remains the only biomarker to predict disease progression in newly diagnosed CP patients [21,37,38]. We interpret our findings within prior functional studies supporting CIP2A–PP2A–STAT5 regulation of BCL-XL. However, the current trial datasets show only that BCL-XL expression is associated with the treatment response and outcome. In matched diagnostic samples, CIP2A RQ and BCL-XL RQ were not significantly correlated (Supplementary Figure S5). This is consistent with the overall study findings, in which CIP2A was linked to disease progression risk [21], whereas BCL-XL was associated with molecular response outcomes in imatinib-treated patients.

The results from the DESTINY TFR trial samples revealed a significant increase in BCL-XL expression during the de-escalation phase among patients who experienced molecular relapse compared to those who achieved TFR. Notably, BCL-XL expression increased by an average of 5.6-fold at 3 and 6 months after starting the de-escalation phase, prior to the detection of molecular relapse or loss of TFR.

TFR stability has been linked to the quiescence of leukemic stem cells (LSCs), which can persist even in the absence of detectable residual disease. LSCs can escape their quiescent state, causing molecular relapse and posing a barrier to CML cure. Immunological surveillance of LSCs plays a crucial role in achieving and maintaining TFR [49,50,51,52,53]. Once TKI treatment begins, most CML cells are eliminated below a specific threshold, enabling the reconstitution of immune cells that suppress residual LSC proliferation. This may contribute to long-term TFR following TKI treatment cessation and, potentially, LSC eradication leading to a CML cure [12,49,54]. The observed elevation in BCL-XL expression could be explained by the involvement of immunological factors in TFR [12,49,54], and/or the characterisation of LSC [55,56]. BCL-XL is highly expressed and essential for LSC survival [26,30,57]. One interpretation of the DESTINY findings is that elevated BCL-XL expression, due to increased proliferation or self-renewal of residual LSCs after TKI treatment, signals an escape from the quiescence status, leading to molecular relapse.

Finally, we have been able to propose a model in which both CIP2A and BCL-XL could be used as a clinical decision-making tool to prevent patients from progressing to blast crisis and enable them to safely stop treatment and achieve a treatment-free remission. This is an advancement that will benefit patients and clinicians. Future work on this clinical decision-making tool is needed, and we hope to test this in a future clinical trial.

## 4. Materials and Methods

### 4.1. Patients

#### 4.1.1. SPIRIT2 Clinical Trial

In the SPIRIT2 trial, 814 newly diagnosed chronic-phase patients were randomly allocated 1:1 to either imatinib 400 mg or dasatinib 100 mg each once daily. Follow-up was monthly for 3 months, 3-monthly until 12 months, and then 6-monthly. Patients were followed for 5 years [58]. This study focused on the first available 200 (25%) diagnostic samples from the SPIRIT2 entrants plus all subsequent patients who progressed. This resulted in a total of 159 samples available/suitable for study, which included 18 of the 23 patients in the entire trial who progressed to the advanced phase (Table 1A). Of those, 64 samples were excluded due to poor cDNA quality/integrity, insufficient volume, or failure of replicate QC. Endpoint analyses were performed within the BCL-XL-measured cohort after stratification by top vs. bottom quartiles (Supplementary Figure S1). Some endpoint data were missing because they were not recorded for certain patients in the trial dataset (TTF *n* = 4; MR2 *n* = 2; MR3 *n* = 2; MR4 *n* = 3; MR4.5 *n* = 4) (Supplementary Figure S6).

#### 4.1.2. DESTINY Clinical Trial

The DESTINY study of TFR investigated the effect of treatment de-escalation prior to complete cessation and included patients with stable MR3 as well as those with MR4 [39]. Its main findings have been previously reported [39]. For the present analysis, we included only DESTINY participants whose samples were biobanked locally in Liverpool. This Liverpool subset comprised 24 local DESTINY entrants, for whom immunological data have been previously published [59]. BCL-XL expressions were analysable in all 24 samples. In addition, two patients who discontinued TKI therapy outside the DESTINY trial were included (Table 1B).

### 4.2. Sample Collection and Preparation

Peripheral blood mononuclear cells were separated from diagnostic samples by density-dependent centrifugation (Lymphoprep Axis-Shield, Cambridge, UK), washed in RPMI 1640 (BioSera, Nuaille, France), and resuspended in RPMI containing 10% dimethylsulfoxide (DMSO) and 10% foetal calf serum (FCS) (BioSera) at 4 °C. Cells were then cryopreserved. Samples were thawed in RPMI containing 10% FCS and 1% L-glutamine using the dropwise method.

### 4.3. Measurement of BCL-XL Expression by Real-Time PCR Assays

We standardised cDNA samples from SPIRIT2 trial patients to 100 ng, suitable for BCL-XL expression analysis, using Nanodrop2000 (Thermo Scientific, Leicestershire, UK). These samples were diluted with RNase-free H_2_O and mixed in a 20 µL qPCR reaction mixture containing 1× TaqMan™ Universal PCR Master Mix (Thermo Scientific, Cat#4304437) and 20× TaqMan BCL-XL gene expression assay (Thermo Scientific, Hs00236329_m1, Cat# 4331182). This mixture also included a final concentration of 250 nM 6-FAM dye-labelled TaqMan probe and 900 nM of both forward and reverse primers. Real-time PCR was performed on a Stratagene Mx3005P (Agilent Technologies, Santa Clara, CA, USA) using standard TaqMan cycling conditions (95 °C for 10 min; 40 cycles of 95 °C for 15 s and 60 °C for 60 s). Samples were analysed in technical replicates (*n* = 2), and the mean C_T was used for quantification; replicates were accepted if C_T SD ≤ 0.50, and otherwise, the sample was re-run. The relative gene expression (RQ) levels were normalised to the endogenous control, GAPDH (Thermo Scientific, Hs99999905_m1, Cat# 4331182), using the comparative method described by Schmittgen and Livak (2008) [60]. GAPDH was chosen as the endogenous control based on stable C_T values across diagnostic samples, consistent with reference-gene selection recommendations [61]. Control templates were generated from a cDNA pool derived from four healthy volunteers.

### 4.4. Statistical Analysis

To stratify patients by BCL-XL expression, we used a pre-specified extreme-quartile approach, defining low (≤0.08 RQ) and high (≥0.33 RQ) groups based on the bottom and top 25% of values. This approach is supported by established methods for identifying biologically relevant expression differences in heterogeneous populations [62,63], as illustrated in Supplementary Figure S1. As a sensitivity analysis, we also evaluated a median split (high vs. low), presented in Supplementary Figure S7. In Kaplan–Meier plots, *p*-values were determined using the log-rank (Mantel–Cox) test; *p*-values are shown where significant (*p* ≤ 0.05). Analyses were undertaken using the statistical package GraphPad Prism v9.5 (GraphPad, San Diego, CA, USA).

### 4.5. Definitions of Outcome Endpoints

The clinical outcome endpoints used in this study are summarised in Table 2. All listed endpoints were assessed in the SPIRIT2 trial, except molecular relapse, which was evaluated in the DESTINY trial.

## 5. Conclusions

These data on BCL-XL expression from the SPIRIT2 and DESTINY trials presented in this research suggest that BCL-XL expression could serve as a predictor for imatinib patients who will not achieve a molecular response. Additionally, it could function as a biomarker for molecular relapse in CML patients considering stopping TKI treatment and maintaining the TFR status. By bringing both CIP2A and BCL-XL together as part of a biomarker toolkit, we may be able to prevent blast crisis and help patients achieve TFR, thus addressing two of the biggest goals in the management of CML patients.

## Figures and Tables

**Table 1:** Patients’ characteristics tables. (**A**) SPIRIT2 patients and (**B**) DESTINY patients.

**(A) SPIRIT2 Patients**					
**Category**			**Imatinib**	**Dasatinib**	**Total**
Patient#			81	78	159
Median age (range)			54 (34–79)	53 (22–81)	53 (22–81)
**Sex**					
Male			50	31	81
Female			56	22	78
**Sokal Score**					
Low			18	13	31
Intermediate			14	22	36
High			16	14	30
N/A			33	29	62
**Hasford Score**					
Low			21	18	39
Intermediate			14	17	31
High			11	7	18
N/A			35	36	71
**EUropean Treatment and Outcome Study (EUTOS)**					
Low			65	54	119
High			11	12	23
N/A			4	13	17
**EUTOS Long-Term Survival (ELTS) Score**					
Low			26	25	51
Intermediate			12	14	26
High			10	10	20
N/A			33	29	62
**(B) DESTINY Patients**					
**Patient Number**	**Age at Entry**	Molecular Relapse **	**TKI**	**DESTINY Subgroup**	
1	65	No	nilotinib	MR4	
2	49	Yes	imatinib	MMR	
3	66	No	imatinib	MR4	
4	57	Yes	imatinib	MR4	
4	58	No	imatinib	MR4	
6	76	No	imatinib	MR4	
7 *	N/A	No	imatinib	N/A	
8	47	No	imatinib	MR4	
9	50	No	nilotinib	MR4	
10	54	Yes	imatinib	MR4	
11	32	Yes	imatinib	MR4	
12	62	No	imatinib	MMR	
13	52	Yes	dasatinib	MR4	
14 *	N/A	Yes	n/a	n/a	
15	62	No	nilotinib	MR4	
16	72	Yes	imatinib	MR4	
17	55	No	imatinib	MR4	
18	67	Yes	dasatinib	MMR	
19	68	No	nilotinib	MR4	
20	43	Yes	imatinib	MR4	
21	62	No	nilotinib	MR4	
22	65	Yes	imatinib	MR4	
23	72	Yes	imatinib	MR4	
24	54	Yes	dasatinib	MR4	
25	53	No	imatinib	MMR	
26	66	Yes	nilotinib	MR4	

# = number N/A = not available. * = stopped treatment outside of the trial. ** = molecular relapse within 14 months of the trial entry.

**Table 2:** Clinical definitions table.

Outcome	Definitions
Overall survival (OS)	Time from trial entry to death from any cause.
Progression-free survival (PFS)	Time from trial entry to disease progression to advanced phase or death from any cause, whichever occurred first.
Freedom from progression (FFP)	Time from trial entry to disease progression alone.
Time to treatment failure (TTF)	Time from trial entry to a change in the allocated therapy because of resistance.
Early molecular response (EMR)	A BCR::ABL1/ABL1^IS^ ratio of ≤10% at 3 months.
Time to molecular response 2 (MR2)	The time to reach a BCR::ABL1/ABL1^IS^ ratio of ≤1%.
Time to molecular response 3 (MR3)	The time to reach a BCR::ABL1/ABL1^IS^ ratio of ≤0.1%.
Time to molecular response 4 (MR4)	The time to reach a BCR::ABL1/ABL1^IS^ ratio of ≤0.01% in the presence of at least 10,000 control ABL1 transcripts.
Time to molecular response 4.5 (MR4.5)	The time to reach a BCR::ABL1/ABL1^IS^ ratio of ≤0.0032% in the presence of at least 31,623 control ABL1 transcripts.
Molecular relapse	Defined as loss of MR3, timed as the first of two consecutive results > 0.1%. Such patients were required to resume their entry TKI at the full standard dose, and were followed monthly until the PCR was ≤0.1%^IS^, at which point they were taken off trial [39].

## Data Availability

The original contributions presented in this study are included in the article/Supplementary Materials. Further inquiries can be directed to the corresponding authors.

## Supplementary Materials

The following supporting information can be downloaded at https://www.mdpi.com/article/10.3390/ijms27072991/s1. Reference [64] is cited in the Supplementary Materials.

## Abbreviations

The following abbreviations are used in this manuscript:

AML	Acute myeloid leukaemia
BCL-2	B-cell lymphoma 2
BCL-XL	B-cell lymphoma-extra-large (anti-apoptotic protein/gene; BCL2L1)
BCR::ABL1	BCR::ABL1 fusion transcript
CIP2A	Cancerous inhibitor of PP2A
CML	Chronic myeloid leukaemia
CP	Chronic phase
DMSO	Dimethylsulfoxide
ELTS	EUTOS long-term survival score
EMR	Early molecular response
EUTOS	European Treatment and Outcome Study
FCS	Foetal calf serum
FFP	Freedom from progression
GAPDH	Glyceraldehyde-3-phosphate dehydrogenase
IS	International Scale
LSC/LSCs	Leukemic stem cell(s)
MMR	Major molecular response
MR2	Molecular response 2
MR3	Molecular response 3
MR4	Molecular response 4
MR4.5	Molecular response 4.5
OS	Overall survival
PCR	Polymerase chain reaction
PFS	Progression-free survival
qPCR	Quantitative polymerase chain reaction
RQ	Relative quantification
RPMI	Roswell Park Memorial Institute medium
S.E.M	Standard error of the mean
STAT5	Signal transducer and activator of transcription 5
TFR	Treatment-free remission
TKI/TKIs	Tyrosine kinase inhibitor(s)
TTF	Time to treatment failure
2G	Second generation
