# Supplementary material for: A Novel CIP2A and BCL-XL Clinical Diagnostic Toolkit to Predict Disease Progression and Treatment-Free Remission in Chronic Myeloid Leukaemia
Source: Int J Mol Sci. 2026 Mar 25;27(7):2991. doi: 10.3390/ijms27072991 (PMC13074176; doi:10.3390/ijms27072991)
Supplement: Supplementary file 1 [file ijms-27-02991-s001.zip › ijms-4112030-supplementary.pdf]

Supplementary Materials: A Novel CIP2A and BCL-XL Clinical Diagnostic Toolkit to Predict Disease Progression and Treatment-Free Remission in Chronic Myeloid Leukaemia

Ammar A. Basabrain [1,2,3,*], Gemma M. Austin [3], Alison K. Holcroft [3], Jane F. Apperley [4], Richard E. Clark [3], Shankar Varadarajan [3] and Claire M. Lucas [3,5,*]

Supplementary Materials

Supplementary Figure S1. (A) Dot plot showing the distribution of baseline BCL-XL mRNA expression levels in SPIRIT2 patients (n = 159), with the lowest quartile (Q1; low BCL-XL) and highest quartile (Q4; high BCL-XL) indicated. **(B)** Table showing the distribution of patients in the low (Q1) and high (Q4) BCL-XL quartiles by treatment arm (imatinib vs dasatinib).

Supplementary Figure S2. Kaplan–Meier curves for overall survival (OS), progression-free survival (PFS), and freedom from progression (FFP) in SPIRIT2 patients stratified by baseline BCL-XL mRNA expression (all patients, imatinib-treated, and dasatinib-treated cohorts).

Supplementary Figure S3. Time to MR2 and MR3 by baseline BCL-XL expression quartiles, including the intermediate group (Q2–Q3): **(A)** dot plot showing quartile distribution and **(B)** Kaplan–Meier curves for the overall cohort and by treatment arm.

Supplementary Figure S4. Kaplan–Meier curves for time to deep molecular response (MR4 and MR4.5) in SPIRIT2 patients stratified by baseline BCL-XL mRNA expression.

Supplementary Figure S5. Correlation between CIP2A RQ and BCL-XL RQ in matched patient samples (scatter plot with Spearman correlation analysis).

Supplementary Figure S6. CONSORT-style flow diagram of SPIRIT2 sample selection and derivation of the BCL-XL analytical cohort, including exclusions, quality-control failures, quartile stratification, and missing endpoint data.

Supplementary Figure S7. Sensitivity analysis using a median cut-off for BCL-XL expression (high vs low), including Kaplan–Meier curves for survival and molecular response endpoints and a dot plot showing median-based group distribution.

Supplementary Table S1. Early molecular response (EMR) in SPIRIT2 patients with high vs low BCL-XL expression (overall cohort and by treatment arm).

Supplementary Table S2: Multivariable Cox Regression Analysis for Time to Treatment Failure, MR2, and MR3 Stratified by BCL-XL Expression

Supplementary Table S3. Clinical and treatment details of 13 molecular relapse patients in the DESTINY cohort.

Supplementary Table S4. (A) Examples illustrating the impact of platelet count on CML risk classification across scoring systems. **(B)** Calculation formulas and risk-group thresholds for Sokal, Hasford, EUTOS, and ELTS scores.

Supplementary Figure S1

A)

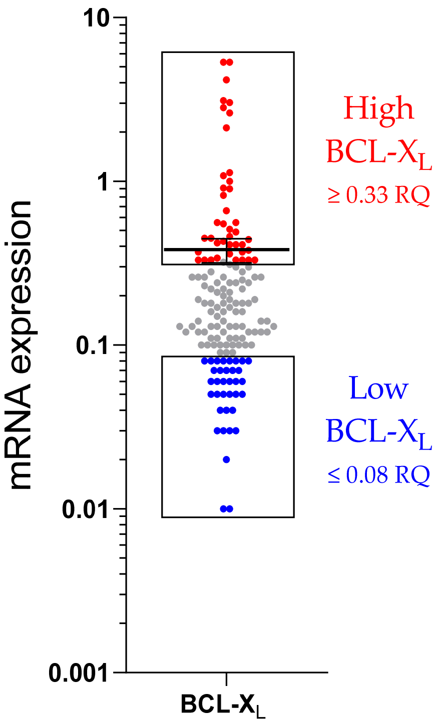

B)

	Imatinib	Dasatinib	Total
**Low BCL-XL**	**19**	**22**	41
**High BCL-XL**	**19**	**22**	41
**Total**	38	44	82

Supplementary Figure S1: A) Dot plot illustrates the distribution of BCL-XL mRNA expression levels among all 159 SPIRIT 2 patients. The high and low BCL-XL expression groups are identified in the highest ((top 25%, ≥ 0.33 RQ, depicted in red) and lowest (bottom 25%, ≤ 0.08 RQ, depicted in blue) quartiles, respectively. B) Table distribution of SPIRIT 2 patients, based on the highest and lowest BCL-XL mRNA expression quartiles as indicated in part A, along with the type of treatment administered to each group.

Supplementary Figure S2

Overall survival (OS)

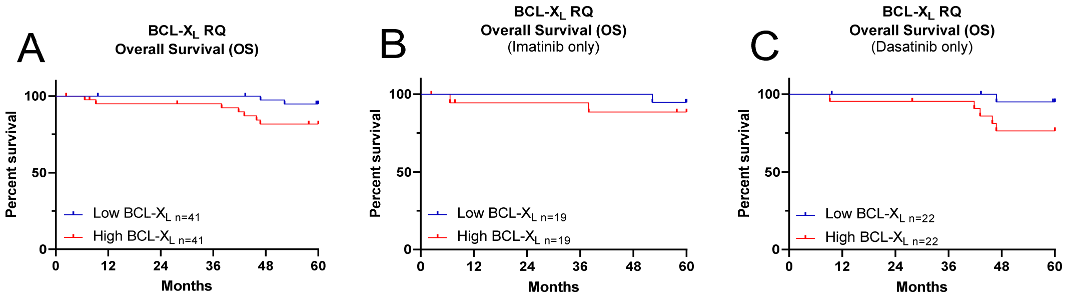

Progression-free survival (PFS)

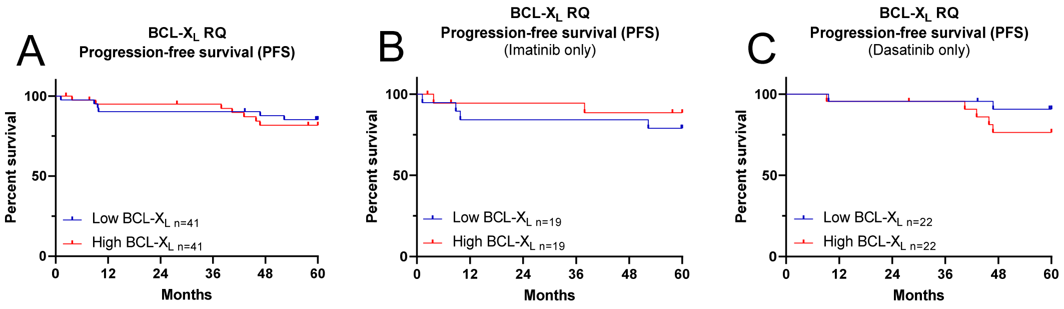

Freedom-from progression (FFP)

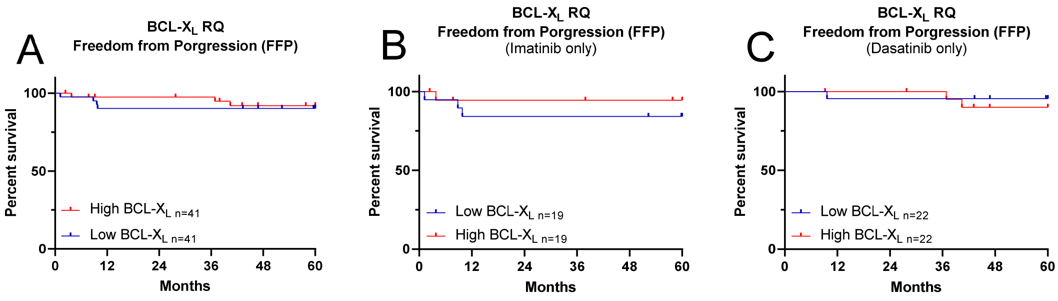

Supplementary Figure S2: Kaplan-Meier curves illustrating the overall survival (OS) in section 2.1, progression-free survival (PFS) in section 2.2, and freedom from progression (FFP) in section 2.3 for SPIRIT2 CML patients, stratified by diagnostic BCL-XL mRNA expression levels. A) Comparison of high and low BCL-XL mRNA expression among all SPIRIT2 CML patients who were administered either imatinib or dasatinib at the time of diagnosis. B) Comparison of high and low BCL-XL mRNA expression levels in SPIRIT2 CML patients who were administered imatinib only, and C) those who were administered dasatinib only

Supplementary Figure S3:
Part A)

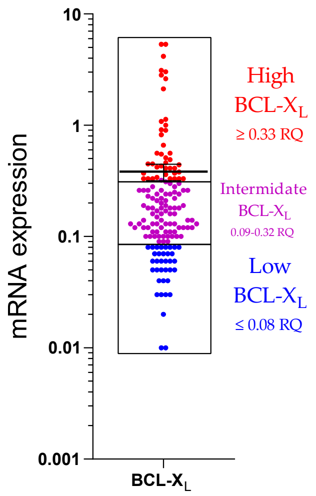

Part B)

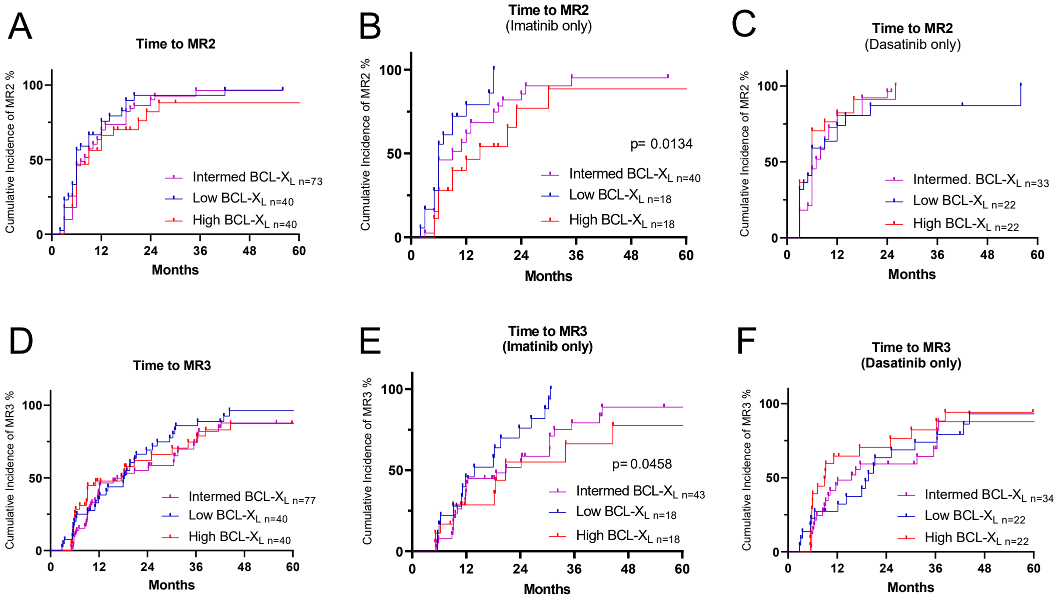

Supplementary Figure S3. Time to MR2 and MR3 by baseline BCL-XL expression quartiles, including the intermediate group (Q2–Q3).

Part A) Dot plot illustrates the distribution of BCL-XL mRNA expression levels among all 159 SPIRIT2 patients. The high and low BCL-XL expression groups correspond to the highest (top 25%; ≥ 0.33 RQ; red) and lowest (bottom 25%; ≤ 0.08 RQ; blue) quartiles, respectively. The intermediate group (Q2–Q3; > 0.08 to < 0.33 RQ; purple) comprises patients between these quartile cutoffs**. Part B)** Kaplan–Meier curves show time to MR2 (A–C) and MR3 (D–F) stratified by baseline BCL-XL expression quartiles: Low (Q1), Intermediate (Q2–Q3), and High (Q4). Panels A and D show the overall cohort Panels B and E show imatinib-treated patients, and panels C and F show dasatinib-treated patients. Tick marks indicate censoring, and p values are from log-rank tests comparing time-to-response across BCL-XL groups within each panel. This supplementary analysis was performed to include patients outside the pre-specified extreme-quartile comparison (Q1 vs Q4). In the overall cohort, the intermediate group showed response kinetics intermediate to the low and high quartiles for both MR2 and MR3. After stratification by treatment, imatinib-treated patients showed significant differences across BCL-XL groups for time to MR2 (p=0.0134, panel B) and MR3 (p=0.0458, panel E), whereas dasatinib-treated patients showed no clear separation for MR2 or MR3 (panels C and F). Time-to-response was analyzed using Kaplan–Meier methods with censoring and log-rank testing

Supplementary Figure S4:

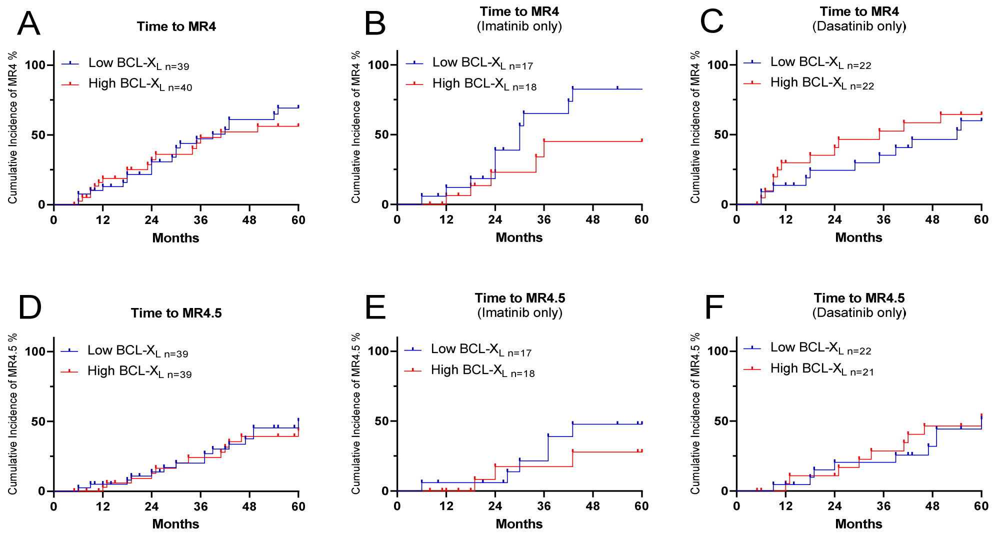

Supplementary Figure S4: Kaplan-Meier curves illustrating MR4 and MR4.5
Cumulative incidence of time to molecular responses (MR4 and MR4.5) for SPIRIT2 CML patients, stratified by their diagnostic BCL-XL mRNA expression levels. Panels (A–C) show Kaplan–Meier curves for time to MR4, while panels (D–F) show Kaplan–Meier curves for time to MR4.5.

Supplementary Figure S5:

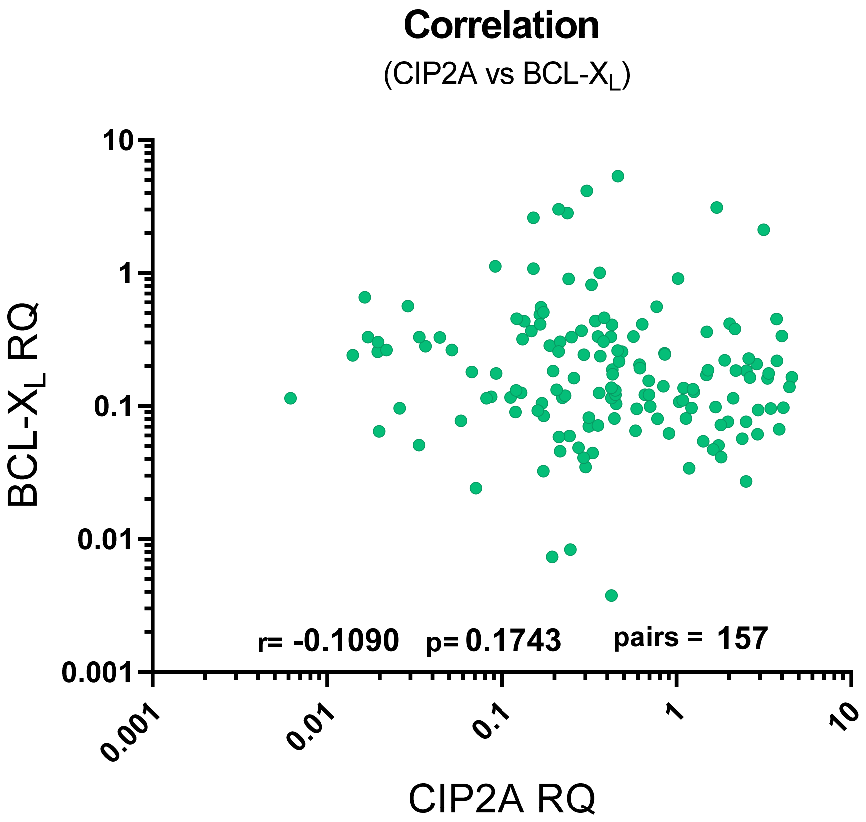

Supplementary Figure S5. Correlation between CIP2A RQ and BCL-XL RQ.

Scatter plot of CIP2A RQ versus BCL-XL RQ in matched patient samples (n = 157). Spearman correlation showed no significant association (r = -0.1090, 95%; p = 0.1743). Axes are shown on a log scale

Supplementary Figure S6:

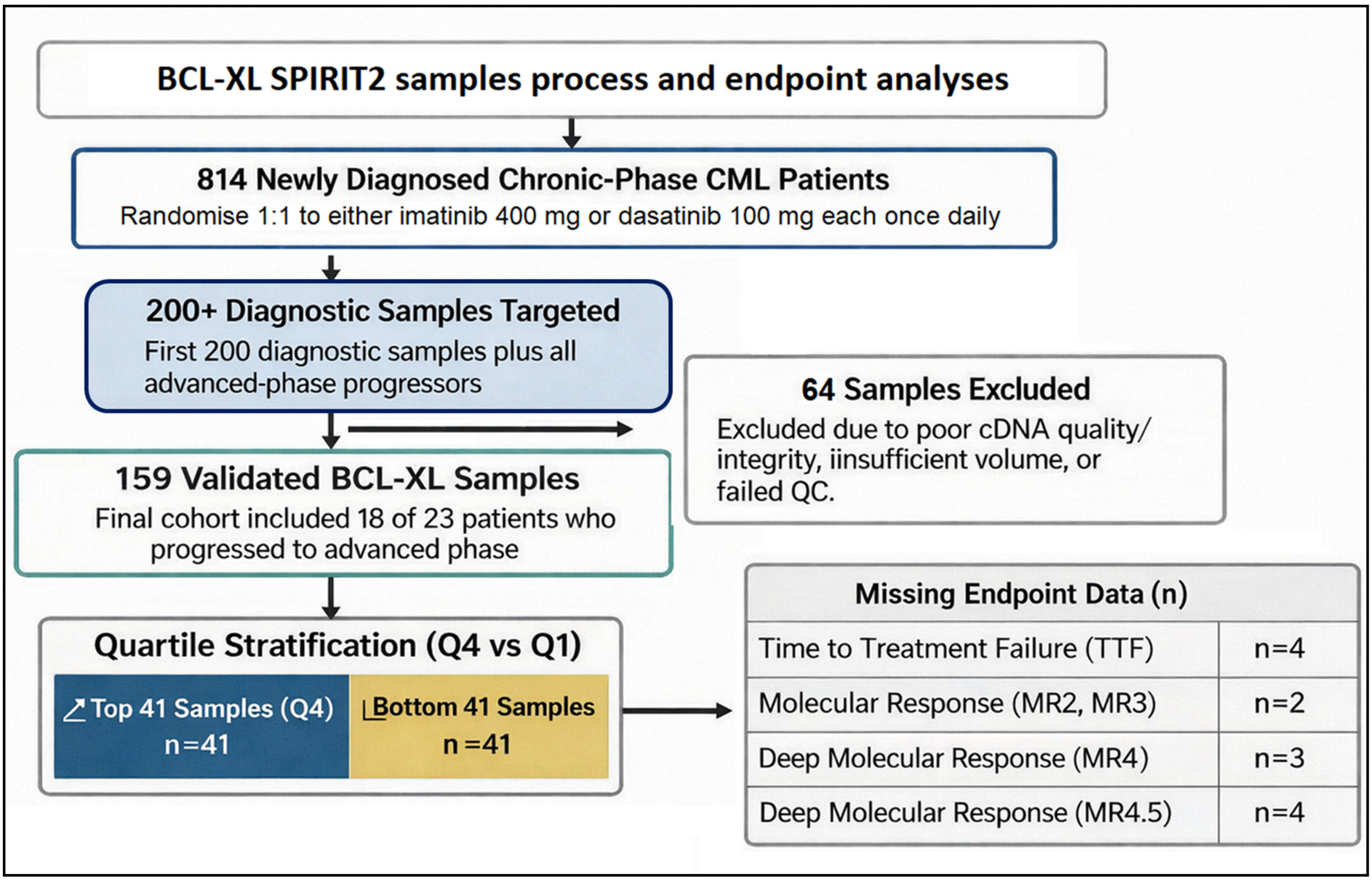

Supplementary Figure S6. SPIRIT2 sample selection process and endpoint analysis cohort for BCL-XL expression. CONSORT-style flow diagram showing derivation of the SPIRIT2 analytical cohort used for BCL-XL mRNA assessment. The parent SPIRIT2 trial included 814 newly diagnosed chronic-phase CML patients randomized 1:1 to imatinib 400 mg once daily or dasatinib 100 mg once daily. For this analysis, 200 diagnostic samples were targeted (the first 200 available diagnostic samples plus later chronic-phase progressors), of which 159 samples passed quality control and were validated for BCL-XL measurement. 64 samples were excluded because of poor cDNA quality/integrity, insufficient sample volume, or failed replicate quality control. The final BCL-XL–measured cohort included 18 of 23 patients who later progressed to advanced phase. For quartile-based endpoint analyses, patients were stratified into top quartile (High; n = 41) and bottom quartile (Low; n = 41) BCL-XL expression groups. Missing endpoint data within the quartile analysis subsets are also shown: time to treatment failure (TTF), n = 4; molecular response (MR2/MR3), n = 2; deep molecular response (MR4), n = 3; deep molecular response (MR4.5), n = 4

Supplementary Figure S7:
A)

Endopoint analysis by Median Cutoff

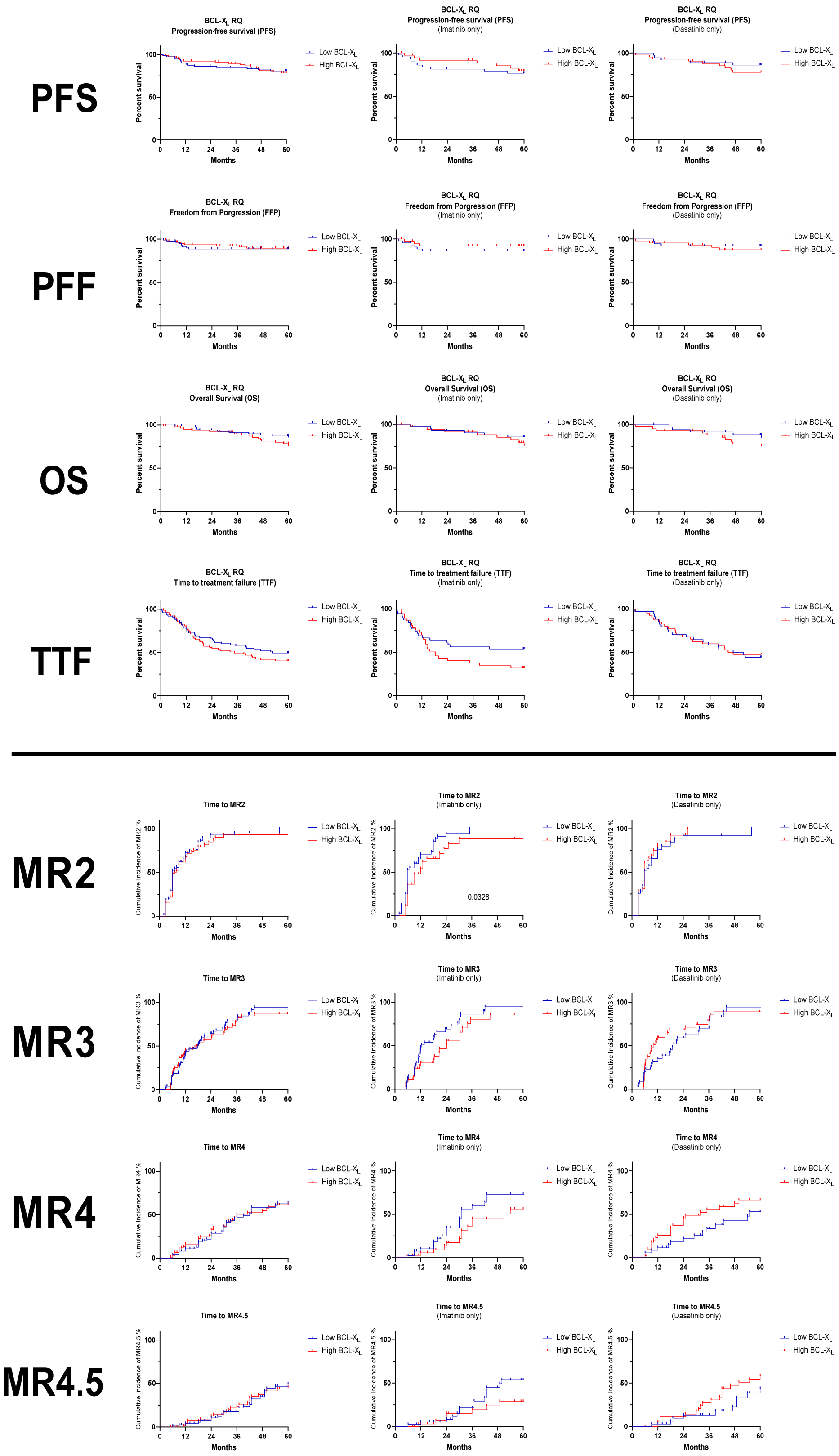

B)

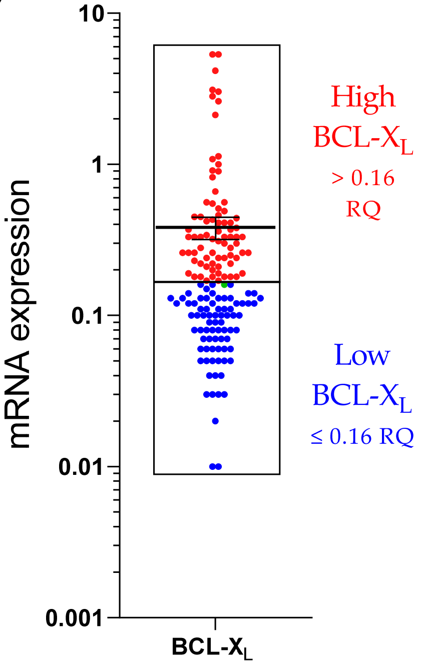

Supplementary Figure S7. Sensitivity analysis using a median cut-off for BCL-XL expression (high vs low). Baseline BCL-XL mRNA was dichotomized at the median (≤ 0.16 RQ vs > 0.16 RQ) as a sensitivity analysis to the pre-specified extreme-quartile comparison (Q1 vs Q4). Kaplan–Meier curves are shown for PFS, PFF, OS, and TTF (upper panel) and time to MR2, MR3, MR4, and MR4.5 (lower panel), for the overall cohort and by treatment arm (imatinib and dasatinib), as labeled. Tick marks indicate censoring; group differences were assessed using the log-rank test. Overall, the median split showed weaker separation than the extreme-quartile approach. B) Dot plot illustrating the distribution of BCL-XL mRNA expression levels among all 159 SPIRIT2 patients. Patients were stratified using a median cut-off into high BCL-XL (> 0.16 RQ, red) and low BCL-XL (≤ 0.16 RQ, blue) groups

Supplementary Table S1: Early Molecular Response (EMR) for SPIRIT2 patient with high and low BCL-XL

Early Molecular Response (EMR)							
	**Low BCL-XL**			**High BCL-XL**			**P Value**
	**Patient #, %**			**Patient #, %**			
	**All patients**						
						(n=74)	0.1
**Not Reach EMR**	13	18%		19	26%		
**Reach EMR**	26	35%		16	22%		
	**Imatinib**						
						(n=33)	**0.033**
**Not Reach EMR**	7	21%		11	34%		
**Reach EMR**	12	36%		3	9%		
	**Dasatinib**						
						(n=41)	0.744
**Not Reach EMR**	6	15%		17	17%		
**Reach EMR**	15	37%		13	32%		

Supplementary Table S2. Multivariable Cox Regression Analysis for Time to Treatment Failure, MR2, and MR3 Stratified by BCL-XL Expression

**Endpoint / Variable**	**Multivariable**		**Multivariable (Imatinib only)**		**Multivariable (Dasatinib only)**	
	**HR (95% CI)**	**p-value**	**HR (95% CI)**	**p-value**	**HR (95% CI)**	**p-value**
**Time to Treatment Failure**						
BCL-XL (High vs Low)	**2.51 (1.34–4.70)**	**0.004**	**4.03 (1.67–9.73)**	**0.002**	1.58 (0.66–3.79)	0.306
Treatment (Dasatinib vs Imatinib)	0.58 (0.32–1.05)	0.073	-	-	-	-
Age (per year)	1.00 (0.98–1.03)	0.87	1.00 (0.97–1.04)	0.876	1.01 (0.98–1.05)	0.438
Gender (Female vs Male)	0.96 (0.51–1.80)	0.902	1.42 (0.59–3.41)	0.432	0.75 (0.31–1.80)	0.518
**MR2**						
BCL-XL (High vs Low)	**1.92 (1.15–3.21)**	**0.013**	**2.78 (1.41–5.48)**	**0.003**	1.28 (0.63–2.61)	0.495
Treatment (Dasatinib vs Imatinib)	1.24 (0.75–2.05)	0.401	-	-	-	-
Age (per year)	1.01 (0.99–1.03)	0.215	1.02 (0.99–1.05)	0.147	1.00 (0.97–1.03)	0.886
Gender (Female vs Male)	1.07 (0.63–1.82)	0.8	0.92 (0.45–1.88)	0.823	1.31 (0.60–2.87)	0.495
**MR3**						
BCL-XL (High vs Low)	**1.73 (1.05–2.84)**	**0.03**	**2.26 (1.16–4.41)**	**0.017**	1.33 (0.65–2.71)	0.437
Treatment (Dasatinib vs Imatinib)	1.10 (0.67–1.80)	0.705	-	-	-	-
Age (per year)	1.00 (0.98–1.02)	0.873	1.01 (0.98–1.04)	0.52	0.99 (0.96–1.02)	0.494
Gender (Female vs Male)	1.25 (0.75–2.09)	0.397	1.17 (0.59–2.32)	0.66	1.34 (0.62–2.91)	0.46

Supplementary Table S3: Details of 13 molecular relapse DESTINY patients*

DESTINY sub-group	*Number of patients*	*%*
MMR	*2*	*17%*
MR4	*10*	*83%*
**TKIs**		
imatinib	*8*	*67%*
dasatinib	*3*	*25%*
nilotinib	*1*	*8%*

*= one molecular relapse patient from outside of the trial

Supplementary Table S4: A) Examples illustrating the impact of patient platelet count on the determination of risk according to various scoring systems, along with the respective calculation formulas in B.

A)

Sokal score	Example 1	Example 2	Different in Platelet
**Age:**	50 years	50 years	
**Spleen size**	3 cm	3 cm	
**% Myeloblasts:**	2%	2%	
**Platelet count:**	100 x 10^3/ µl	1580 x 10^3/ µl	1580-100 = 1480
**Patient's outputs**			
**Patient's Sokal Score:**	0.8	1.8	
**Risk Group:**	Intermediate	High	increase in platelet count cause increased in the risk value

Hasford score	Example 1	Example 2	Different in Platelet
**Age:**	40 years	40 years	
**Spleen size**	5 cm	5 cm	
**% Myeloblasts:**	3%	3%	
**% Eosinophils**	3%	3%	
**% Basophils**	3%	3%	
**Platelet count:**	100 x 10^3/µl	1580 x 10^3/ µl	1580-100 = 1480
**Patient's outputs**			
**Patient's Hasford Score:**	713.00	1808.60	
**Risk Group:**	Low	High	increase in platelet count cause increase in the risk value
**Hasford score range**	≤ 780	≥ 1480	

ELTS score	Example 1	Example 2	Different in Platelet
**Age:**	50 years	50 years	
**Spleen size**	5 cm	5 cm	
**% Blasts:**	3%	3%	
**Platelet count:**	100 x10^3/µl	1580 x10^3/µl	1580-100 = 1480
**Patient's outputs**			
**Patient's ELTS score:**	2.23	1.26	
**Risk Group:**	High	Low	increase in platelet count cause decrease the risk value
**ELTS score range**	> 2.2185	≤ 1.5680	

B)

Establishment, calculations formula and risks groups of predictive scoring systems in CML. Adapted from [1]

	**Calculations Formula**	**Risk groups**
**Sokal score**<br>**1984**<br><br>**813 patients treated with chemotherapy**	Sokal score=<br>16× (age [in years]−43.4)<br>+ 0.0345× (spleen size [cm below costal margin]−7.51)<br>+ 0.1880× ((platelet count [in 10^9/L]/700)^2−0.563)<br>+ 0.0887× (blasts [% in peripheral blood] −2.10)	Low risk: <0.80<br><br>Intermediate risk: ≥0.80 and ≤1.20<br><br>High risk: >1.20
**Hasford score**<br>**1998**<br><br>**1573 patients treated with interferon-alpha**	HASFORD score=<br>(0.6666×age [0 when age <50 years; 1, otherwise]<br>+ 0.0420×spleen size [cm below costal margin]<br>+ 0.0584×blasts [% in peripheral blood]<br>+ 0.0413×eosinophils [% in peripheral blood]<br>+ 0.2039×basophils [0 when basophils [% in peripheral blood] <3;1, otherwise]<br>+ 1.0956×platelet count [0 when platelets count [in 10^9/L] <1,500;1, other-wise])×1,000	Low risk: ≤780<br><br>Intermediate risk: >780 and ≤1,480<br><br>High risk: >1,480
**EUTOS score**<br>**2011**<br>**2060 patients treated with imatinib**	EUTOS score =<br>7×basophils [% in peripheral blood]<br>+ 4×spleen size [cm below costal margin]	Low risk: ≤87<br><br>High risk: >87
**ELTS score**<br>**2015**<br>**2290 patients treated with imatinib**	ELTS score=<br>0.0025 x (age in completed years/10)^{3}.<br>+ 0.0615 x spleen size below costal margin<br>+ 0.1052 x blasts in peripheral blood<br>+ 0.4104 x (platelet count/1000)$^{-0.5}$	Low risk: ≤ 1.5680<br><br>Intermediate risk: > 1.568 and ≤ 2.2185<br><br>High risk:> 2.2185

References

1. Pfirrmann, M.; Lauseker, M.; Hoffmann, V. S.; Hasford, J., Prognostic Scores for Patients with Chronic Myeloid Leukemia Under Particular Consideration of Disease-Specific Death. In *Chronic Myeloid Leukemia*, Springer: 2021; pp 119-143.
